# Ecological momentary assessment (EMA) combined with unsupervised machine learning shows sensitivity to identify individuals in potential need for psychiatric assessment

**DOI:** 10.1007/s00406-023-01668-w

**Published:** 2023-09-16

**Authors:** Julian Wenzel, Nils Dreschke, Esther Hanssen, Marlene Rosen, Andrej Ilankovic, Joseph Kambeitz, Anne-Kathrin Fett, Lana Kambeitz-Ilankovic

**Affiliations:** 1 Department of Psychiatry and Psychotherapy, Faculty of Medicine and University Hospital of Cologne, University of Cologne, Cologne, Germany; 2 Hersencentrum Mental Health Institute, Amsterdam, The Netherlands; 3 Department of Psychiatry, Faculty of Medicine, University of Belgrade, Belgrade, Serbia; 4 Department of Psychology, City, University of London, London, UK; 5 Department of Psychosis Studies, Institute of Psychiatry, Psychology and Neuroscience, King’s College London, London, UK; 6 Faculty of Psychology and Educational Sciences, Department of Psychology, Ludwig-Maximilian University, Munich, Germany

**Keywords:** EMA, Psychosis, Clustering, Dynamic time warping, Unsupervised machine learning

## Abstract

Ecological momentary assessment (EMA), a structured diary assessment technique, has shown feasibility to capture psychotic(-like) symptoms across different study groups. We investigated whether EMA combined with unsupervised machine learning can distinguish groups on the continuum of genetic risk toward psychotic illness and identify individuals with need for extended healthcare. Individuals with psychotic disorder (PD, *N* = 55), healthy individuals (HC, *N* = 25) and HC with first-degree relatives with psychosis (RE, *N* = 20) were assessed at two sites over 7 days using EMA. Cluster analysis determined subgroups based on similarities in longitudinal trajectories of psychotic symptom ratings in EMA, agnostic of study group assignment. Psychotic symptom ratings were calculated as average of items related to hallucinations and paranoid ideas. Prior to EMA we assessed symptoms using the Positive and Negative Syndrome Scale (PANSS) and the Community Assessment of Psychic Experience (CAPE) to characterize the EMA subgroups. We identified two clusters with distinct longitudinal EMA characteristics. Cluster 1 (*N*_PD_ = 12, *N*_RE_ = 1, *N*_HC_ = 2) showed higher mean EMA symptom ratings as compared to cluster 2 (*N*_PD_ = 43, *N*_RE_ = 19, *N*_HC_ = 23) (*p* < 0.001). Cluster 1 showed a higher burden on negative (*p* < 0.05) and positive (*p* < 0.05) psychotic symptoms in cross-sectional PANSS and CAPE ratings than cluster 2. Findings indicate a separation of PD with high symptom burden (cluster 1) from PD with healthy-like rating patterns grouping together with HC and RE (cluster 2). Individuals in cluster 1 might particularly profit from exchange with a clinician underlining the idea of EMA as clinical monitoring tool.

**Supplementary Information:**

The online version contains supplementary material available at 10.1007/s00406-023-01668-w.

## Introduction

Ecological momentary assessment (EMA), a structured diary assessment technique, has shown feasibility to capture psychotic symptoms in daily life [1, 2]. Its good construct validity in capturing and linking symptoms between EMA and established clinical questionnaires has previously been demonstrated [3] and EMA ratings of psychotic symptoms can differentiate individuals on the psychosis spectrum [4–6]. EMA is self-administered, reflects high ecological validity, and delivers a longitudinal perspective on (subtle) experiences of symptoms [7]. Thus, it represents a potentially useful tool for monitoring mental health conditions [3, 8, 9]. However, to the best of our knowledge, no study yet has investigated whether unsupervised machine learning (ML) can distinguish groups on the continuum of genetic risk toward psychotic illness [10] and identify individuals with potential need for extended healthcare based on their longitudinal trajectories of psychotic(-like) experiences in EMA.

Many individuals who have experienced a first psychotic episode are likely to experience relapse in symptoms [11]. EMA represents a cost-efficient and ecologically sensitive way of monitoring psychotic symptoms, might support clinicians in identifying individuals with increased risk of relapse and meets the need of individuals with lived experience of psychosis to monitor their symptoms [3, 8]. Therefore, it is important to understand the sensitivity of EMA to identify individuals in need of in person psychiatric assessment in situations where their current condition is not known, e.g., in out-clinic patients. More specifically, it is of interest to investigate how bottom-up unsupervised ML algorithms, that work agnostic of study group assignment, cluster healthy individuals and individuals with different degrees of psychotic symptoms based on similarities in their longitudinal symptom pattern in EMA.

Though research using top-down statistical approaches indicates that “knowing” the individual study groups, EMA is discriminative with respect to psychotic and subtle psychotic-like experiences, such groups are potentially less distinct than expected. On the one hand, individuals with psychotic disorders show variability in reporting psychotic symptoms in EMA as in a previous study only a fraction of the psychotic individuals reported symptoms in the form of visual and auditory hallucinations [1]. Additionally, due to the perception of being “monitored” through EMA, individuals experiencing persecutory ideas might hesitate to interact with the app [12]. On the other hand, psychosis-like experiences also occur in the general population in different severities, potentially becoming clinically relevant [10, 13]. They have been reported in healthy individuals as observed in large-scale epidemiological surveys [14–16]. In individuals at clinical high-risk for psychosis, psychotic-like experiences can temporarily surpass the clinical threshold [17]. The ecological and intensive longitudinal nature of EMA might be particularly suitable to observe more subtle psychotic-like phenomena.

A challenge in the analysis of EMA data is the fact that study participants typically provide symptom ratings at different times of the day and with different time intervals between measurements. Dynamic time warping (DTW) uses “stretching” and “compression” to align two time series, has been commonly applied in speech recognition [18] and only recently in psychiatric research to assess temporal similarities in symptom clusters of depressive patients [19]. This technique might account for temporal delays in EMA ratings and captures similarities in the overall pattern of ratings where a time point per time point comparison might reveal low consistency.

In this proof-of-concept study we used EMA combined with unsupervised ML to examine the extent to which longitudinal trajectories of psychotic(-like) experiences in EMA are distinctive between groups on the continuum of genetic risk toward psychotic illness. Further we investigated whether this approach shows the potential to identify a subgroup of individuals vulnerable to relapse into psychotic illness to provide evidence for the implementation of EMA as digital mental healthcare device in clinical practice. Outpatients diagnosed with a psychotic disorder (PD), healthy individuals (HC), and healthy individuals with a first-degree relative with psychosis (RE) were recruited as part of two previous projects, the DECOP study [20] and the SMARTAPP study [21]. Over a period of 7 days, we analyzed ratings on a psychotic symptom scale which we calculated as the average of questionnaire items related to auditory and visual hallucinations and paranoid ideation and which have shown high internal consistency and good construct validity in previous studies [22–25]. We (1) combined unsupervised ML with DTW to cluster the psychotic symptom ratings of each individual based on longitudinal characteristics, i.e., similarities in rating intensity and rating variance over the EMA period, agnostic of study group assignment. Further, we 2) characterize the obtained subgroups with respect to clinical assessments administered prior to EMA and 3) evaluate the correspondence between participant’s EMA symptom ratings and the original study group assignment.

## Methods

### Sample

PD, RE and HC were recruited through multiple clinical services, including community treatment teams, hospitals, patient- and relative associations, NHS foundation trusts, research collaborators and online advertising as described in detail in previous studies [20, 21, 26, 27]. The sample of the DECOP study (PD = 34, HC = 27, RE = 21) was acquired in the United Kingdom and the sample of the SMARTAPP study in the Netherlands (PD = 64). The studies received ethical approval from the medical research ethics committee of the Medical Center of the VU University Amsterdam [NL56511.068.16] and the London-Harrow Research Ethics Committee [14/LO/0710], respectively. For the analysis data was pooled across both samples (Table 1, see supplementary material and table S1 for sample comparison).

**Table 1:** Demographic and clinical sample characteristics

	HC	PD	RE	Statistics	
	*N* = 25	*N* = 55	*N* = 20	F (2,97)/X^2^(2, 100)	p value
Mean age (SD)	36.4 (8.2)	39.9 (10.3)	37.2 (14.7)	1.056	0.352
Gender (female, %)	9 (36.0)	16 (41.0)	14 (70.0)	10.444	< 0.01
Living status (%)				18.901	< 0.001
Alone	28.0	67.3	20.0		
Family/partner	48.0	23.7	60.0		
Other	24.0	9.0	20.0		
Diagnosis (%)					
Psychotic disorder	–	11.6	–		
Schizoaffective Disorder	–	21.1	–		
Schizophrenia	–	67.3	–		
PANSS score (SD)					
General	–	29.6 (7.2)	–		
Positive	–	14.2 (5.5)	–		
Negative	–	15.3 (5.6)			
Medication, n (%)^a^					
Antipsychotics	–	49 (96.1%)	–		
Antidepressants	–	15 (29.4%)	–		
Benzodiazepines	–	5 (9.8%)	–		
Mood stabilizers	–	1 (2.0%)	–		

*HC* healthy controls;* PD* individuals with psychotic disorder;* RE* healthy relatives of individuals with psychotic disorder; *PANSS* positive and negative syndrome scale

^a^information on medication is based on N=51 PD individuals

The inclusion criteria for participants across both studies were 1) age between 18 and 60 years, 2) intelligence quotient > 70 and 3) ability to read and understand the English/Dutch language. The exclusion criteria for all participants in the DECOP study were history of neurological illness or diagnosis of alcohol/drug dependence no longer than 6 months prior to study screening. Specific inclusion criteria for outpatients were a diagnosis of schizophrenia according to the Diagnostic and Statistical Manual of Mental Disorders (DSM-5) (SMARTAPP study) or non-affective psychosis according to ICD-10 criteria with stable pharmacological treatment (> 6 weeks) at the time of inclusion (DECOP study). RE were recruited solely within the DECOP study and had to be unrelated to outpatients included in the study. All authors confirmed that procedures related to the current work comply with ethical standards of the relevant institutional committees on human experimentation and with the Helsinki Declaration of 1975, as revised in 2008. All participants included in the studies provided a written informed consent.

From the 82 participants of the DECOP sample, six individuals (4 PD, 1 RE, 1 HC) were excluded from the analysis as they reacted to less than one-third of the presented beeps [28]. Additionally, one HC was excluded due to antidepressant intake and one participant could not be included due to technical problems with the app. Therefore, the final DECOP analysis data set consisted of 74 participants (PD = 29, HC = 25, RE = 20). From the 64 individuals of the SMARTAPP sample, 11 individuals were excluded due to a variety of reasons including, wrong diagnosis, technical problems in data transmission, non-completion due to personal reasons and fewer than 30% of the data points in the EMA questionnaires filled out [28]. We used the data of 26 individuals randomly assigned to the no-feedback arm of the study.

### Ecological-momentary assessment (EMA)

Individual trajectories of symptoms were recorded using EMA as described in detail previously [20, 21]. The EMA questionnaire was conducted through the PsyMate™ platform (www.psymate.eu) in the SMARTAPP study and through a custom-made application in the DECOP study. For the DECOP study, each participant was handed an iPod or used their own iPhone for answering the questionnaires. Participants in the SMARTAPP study used their own phone or could borrow a study phone (LG) for the duration of the study. During a period of seven consecutive days, participants were instructed to complete short questionnaires which appeared pseudo-randomly for up to ten times a day between 8:00 am and 10:30 pm in the DECOP study. In the SMARTAPP study participants were alerted up to six times a day to fill questionnaires which appeared pseudo-randomly between 10:00 am to 10:00 pm over three weeks. We used ratings from day 2–8 of the SMARTAPP sample for the current analysis. A 7-day period is typically chosen in EMA as it ensures good compliance at relatively low burden for the participant [7, 29–31]. The EMA questionnaire consisted of 30 items. Participants were prompted with 4 additional questions depending on the answer to the item “I am on my own.” We operationalized psychotic symptoms as the average of the following items that were phrased identically between DECOP and SMARTAPP samples: “I hear voices,” “I see things,” “I feel that others dislike me,” “I feel suspicious,” “I feel that others intend to harm me.” Possible responses for each item were made on a seven-point Likert scale (1 = “not at all” to 7 = “very”). The paranoia items chosen for the current analysis (“I feel suspicious,” “I feel that others intend to harm me,” “I feel that others dislike me”) have shown high internal consistency (Cronbach’s alpha = 0.89) [22, 23] and good construct validity with the paranoia scale (*r* = 0.42, *p* < 0.001) and Positive and Negative Syndrome Scale (PANSS; [32]) item P6 (paranoia/persecution; *r* = 0.58, *p* < 0.001; [22]). In addition, items capturing visual and auditory hallucinations (“I hear voices,” “I see things”) have been significantly associated with the PANSS hallucination score and PANSS positive symptoms score [1]. In sum, the validity of the paranoia items and items related to hallucinations has been shown previously [1, 22–25].

To harmonize the EMA sampling windows of the DECOP (8:00 am to 10:30 pm) and SMARTAPP (10:00 am to 10:00 pm) study, we defined six 2-h time slots per day: before 12:00 am (slot 1), 12:00 am to 2:00 pm (slot2), 2:00 pm to 4:00 pm (slot3), 4:00 pm to 6:00 pm (slot 4), 6:00 pm to 8:00 pm (slot 5) and 8:00 pm to 10:00 pm (slot 6). Scores were averaged if participants provided ratings more than once in a specific time window.

### Symptom measures

Personnel of both study sites assessed psychotic symptoms in outpatients based on the PANSS at the beginning of the study. Psychotic symptoms in outpatients were measured using the PANSS at the beginning of the study. The PANSS questionnaire is an observer rating instrument for quantification of psychotic symptom severity consisting of 30 items which can be assigned to a positive (7 items), negative (7 items) and general symptom scale (16 items) [32]. Further, the Community Assessment of Psychic Experience (CAPE)[33], a self-rated measure of the frequency of and the distress due to positive and negative psychotic(-like) experiences and depression, was conducted to capture such experiences in all study groups.

### Data preprocessing

Missing values of the five extracted items across the six time slots were inspected for the DECOP and SMARTAPP samples separately. The DECOP and SMARTAPP sample contained a total of 27.8% and 46.7% missing values, respectively, over the seven-day period defined for the analysis (supplementary Fig. S1).

We imputed missing values in the EMA data using a random forest-based imputation approach as implemented in the R package missForest [34, 35]. We pooled the SMARTAPP and DECOP sample and imputed missing data by using temporally preceding or following ratings of the same EMA item (e.g., “I hear voices”). In the first step, missing values were imputed by calculating the mean of all values of a given observation within a given item. In the second step, a random forest was built based on the imputed variable. In the third step, the previously imputed values are predicted based on this model. When the predicted values improved the previously imputed values, i.e., the difference between the predicted and previously imputed values were decreasing, the predicted values replaced the previously imputed values. The second and third step were repeated until the error between previously imputed and predicted values started to increase, and the imputed values of the prior iteration were kept as the final result.

We calculated the ‘psychotic symptom rating’ by computing the mean across the imputed values of the five extracted EMA items (“I hear voices,” “I see things,” “I feel that others dislike me,” “I feel suspicious,” “I feel that others intend to harm me”).

### Dynamic time warping and clustering analysis for symptom trajectories

**Fig. 1:**
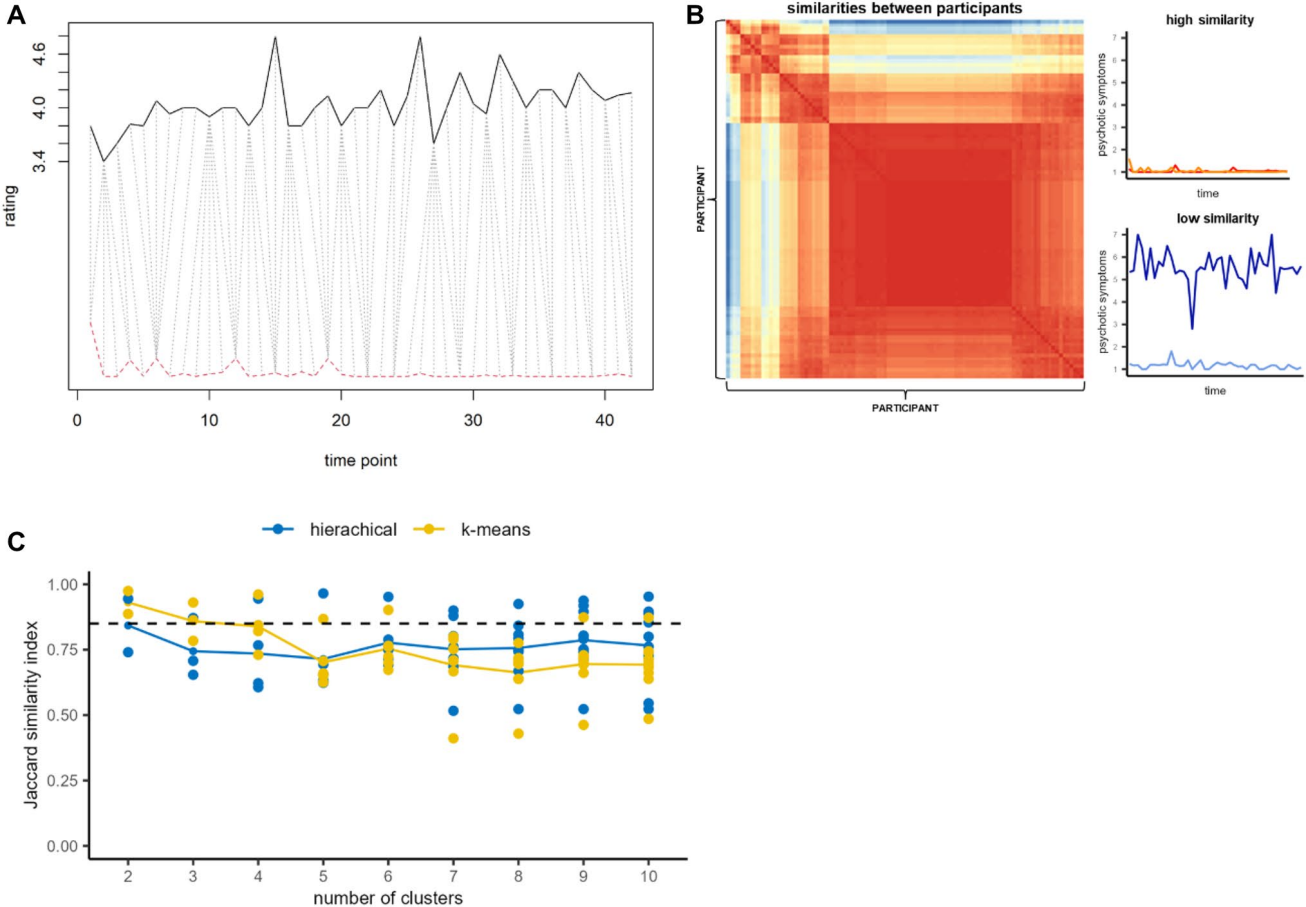
Similarities between symptom trajectories and clustering procedure. **A** Dynamic time warping (DTW) using a symmetric step pattern (“symmetricP0”) is applied to align two symptom trajectories of individuals by stretching and compressing their time series (selected window size = 2). **B** A matrix showing pairwise comparisons of individuals with similar (red) and dissimilar psychotic symptom trajectories is generated. **C** For a pre−defined cluster range of 2 to 10 clusters, hierarchical (blue) and k−means (yellow) cluster algorithms are applied on the similarity matrix within a resampling approach. Jaccard similarity indices above 0.85 (dashed line) indicate highly stable cluster solutions

To evaluate similarities between individual psychotic symptom trajectories across the EMA period, we used DTW as implemented in the R-package “parallelDist” [36] and the R-package “pheatmap” [37] for visualization of distances. Through “stretching” and “compression” two time series were aligned in a way that the Euclidean distance between values of each time series is minimized. The amount of stretching and compressing is regulated by the window size which determines the number of time points in a time series, a given time point in another time series is compared to during alignment. We used a Sakoe Chiba Band [38] with window sizes of two, four, eight and sixteen to assess similarity for different extents of delay in ratings and applied a symmetric step pattern (‘symmetricP0’) [19]. The process resulted in a similarity matrix which represented pairwise distances between symptom trajectories (Fig. 1, supplementary Fig S2).

This similarity matrix was used as a basis for k-means and agglomerative hierarchical clustering. The K-means clustering algorithm is an unsupervised ML approach that partitions observations into K a priori defined subgroups by minimizing their distance to a given cluster centroid [39]. Agglomerative hierarchical clustering starts with each individual observation as a single cluster and iteratively merges close data points/clusters by minimizing their in-group variance (method = “ward.D2”) until a certain criterion is achieved [39]. In the current analysis we conducted both hierarchical agglomerative clustering and k-means clustering, with the aim to compare their performance in terms of cluster stability. We ran both clustering algorithms on cluster numbers between 2 to 10 and assessed cluster stability using the Jaccard similarity indices within a resampling approach based on the function “clusterboot” [40] implemented in the R-package “fpc” [41] (Fig. 1c). A random subset of 50% of the total observations (without replacement) was drawn for *N* = 1000 times for each number of clusters (2 to 10; Fig. 1c) and the clustering algorithm determined the cluster assignments on each individual subset of the data [40]. Subsequently, the Jaccard index [42] was used to obtain a measure of similarity between cluster solutions of each subset, i.e., to assess how often certain individuals are clustered together. This procedure ensured that the cluster solution is not driven by outliers in the data and therefore reduced the chance of overfitting the cluster model to the data set. Additionally, we visually inspected distances between different cluster numbers using a dendrogram (supplementary Fig S2). The decisions on cluster algorithm and final number of clusters for further characterization were based on both the assessment of cluster stability and the interpretation of the dendrogram.

### Statistical comparisons between symptom trajectory clusters and clinical variables

One-factorial Analyses of Variance (ANOVA) were calculated for continuous demographic and clinical measures. PANSS negative, positive, and general scores and specific PANSS items related to the items used for EMA (P1: delusions, P3: hallucinatory behavior, P6: suspiciousness/persecution, N2: emotional withdrawal, G16: active social avoidance) are only compared for PD. One-factorial Analyses of Variance (ANOVA) are calculated to characterize EMA differences between the symptom trajectory clusters. Therefore, we calculated the mean across all ratings and the corrected rating variance (rating variance divided by the mean rating), i.e., is the within-subjects temporal variance, for each cluster.

P-values of the main effects were corrected using false discovery rate (FDR) [43] separately for variables on demographics, cluster characterization and clinical instruments. Post hoc t-tests for significant main effects were conducted and p-values were corrected using FDR. Nominal scales (e.g., sex) and the distribution of study groups across clusters were analyzed using chi-square tests.

All analyses were conducted in R version 4.0.3 (https://cran.r-project.org/bin/windows/base/).

## Results

As indicated by Jaccard indices higher than the critical threshold of 0.85 (Fig. 1c) and a drop in cluster distance in the dendrogram (Fig S2) our clustering procedure for symptom trajectories identifies a two-cluster solution as optimal for the current data set. Varying the window size of the Sakoe Chiba Band, i.e., allowing for more or less stretching and compression, had no impact on the number of clusters identified and only minimal impact on the observations grouped within clusters (supplementary material; supplementary Fig S2). In the following, we represent the cluster characteristics obtained for a window size of two.

**Fig. 2:**
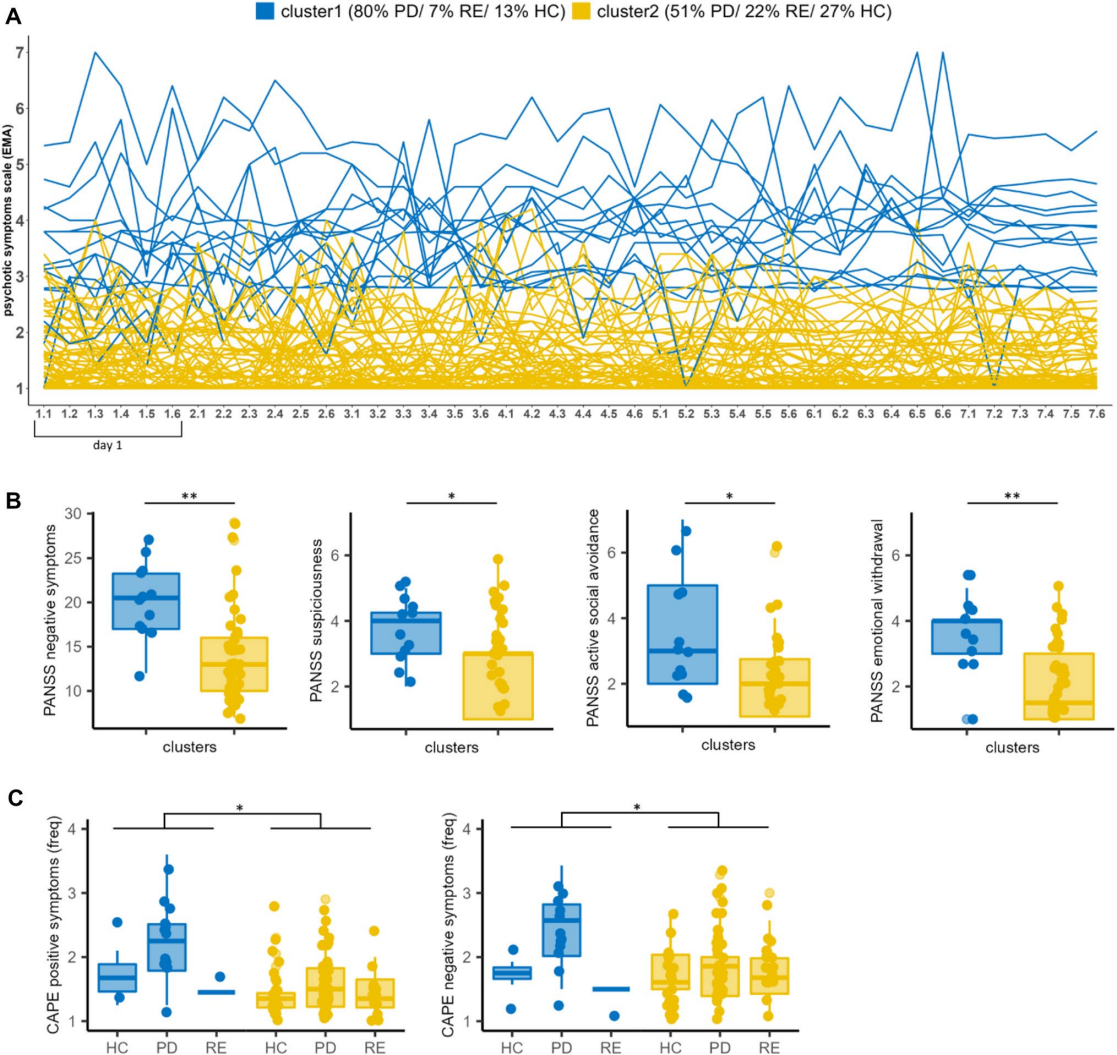
Cluster characteristics. We obtained a two−cluster solution with distinct EMA rating characteristics (**A**). Clusters showed significantly different clinical scores on the PANSS (**B**) and CAPE (**C**) questionnaire. Abbreviations: *PD* = individuals with psychotic disorder, *HC* = healthy controls, *RE* = healthy relatives of individuals with psychotic disorder. Significances: *p < 0.05, **p < 0.01, ***p < 0.001

Cluster 1 (*N* = 15) comprises of 13.3% HC (*N* = 2), 6.7% RE (*N* = 1) and 80.0% PD (*N* = 12) and cluster 2 (*N* = 85) comprises 27.1% HC (*N* = 23), 22.4% RE (*N* = 19) and 50.5% PD (*N* = 43) (Table 2). Cluster 1 mostly contains PD and cluster 2 is more balanced though proportions of study groups between clusters are not significantly different (*X*^2^(2, 100) = 4.53, *p* = 0.161). The clusters hold distinct characteristics with respect to their average EMA psychotic symptom rating across the seven days (*t*(15.66) = 11.25, *p* < 0.001, 95% CI [1.91, 2.80]) showing higher average EMA ratings for cluster 1 than cluster 2. We find no statistical differences with respect to rating variance (*t*(26.00) = 0.80, *p* = 0.43, 95% CI [− 0.03, 0.06]) (Fig. 2a, supplementary Fig S3).

**Table 2:** Demographic and clinical cluster characteristics

	Cluster 1 (*N* = 15)	Cluster 2 (*N* = 85)	t value/chi^2^	p (fdr)
Study group^a^	12/1/2	43/19/23	4.53	0.294
Age, mean (sd)	38.8 (12.1)	38.4 (10.7)	0.11	> 0.999
Sex = female (%)	1 (6.6%)	38 (44.7%)	7.75	0.031*
Educational status^b^	2/2/5/2/0/2	27/19/11/0/2/2	18.00	0.016*
Medication, n (%)^c^				
Antipsychotics	10 (91.0%)	39 (97.5%)	0.99	0.622
Antidepressants	2 (18.2%)	13 (33.3%)	0.94	0.622
Benzodiazepines	0 (0.0%)	5 (13.2%)	1.61	0.622
Mood stabilizers	0 (0.0%)	1 (2.6%)	0.30	> 0.999
PANSS				
Positive symptoms, mean (sd)	16.2 (4.0)	13.6 (5.7)	1.74	0.150
Negative symptoms, mean (sd)	20.3 (4.3)	13.8 (5.1)	4.45	0.001**
General symptoms, mean (sd)	31.6 (6.3)	29.0 (7.5)	1.21	0.240
PANSS (individual items)				
Delusions (P1)	2.8 (1.3)	2.2 (1.3)	1.31	0.234
Hallucinatory behavior (P3)	3.3 (1.8)	2.4 (1.6)	1.59	0.174
Suspiciousness/persecution (P6)	3.7 (1.1)	2.6 (1.4)	2.71	0.031*
Emotional withdrawal (N2)	3.6 (1.1)	2.0 (1.2)	4.40	0.001**
Active social avoidance (A16)	3.5 (1.8)	2.0 (1.2)	2.77	0.031*
CAPE				
Positive symptoms—freq, mean (sd)	2.1 (0.6)	1.5 (0.4)	3.66	0.013*
Positive symptoms—dis, mean (sd)	2.3 (0.9)	1.9 (0.6)	2.10	0.082
Negative symptoms—freq, mean (sd)	2.3 (0.6)	1.8 (0.5)	2.81	0.036*
Negative symptoms—dis, mean (sd)	2.4 (0.6)	2.0 (0.6)	2.04	0.082
Depressive symptoms—freq, mean (sd)	2.2 (0.6)	1.9 (0.6)	1.61	0.150
Depressive symptoms—dis, mean (sd)	2.4 (0.7)	2.5 (0.7)	-0.30	0.765

*freq* frequency;* dis* distress;* sd* standard deviation

^a^numbers correspond to PD/RE/HC

^b^ numbers correspond to university/college/secondary school/primary school/other/none

^c^information on medication is based on N=51 PD individuals

### Demographic differences between clusters

Clusters differ with respect to  sex (*X*^2^(1, 100) = 7.75, *p* < 0.05) and educational status (*X*^2^(2, 100) = 18.00, *p* < 0.05) as cluster 1 consists of mainly male participants with lower educational level. However, educational status is only assessed for the DECOP sample (Table 2).

### Clinical differences between clusters

Relative to cluster 2, cluster 1 shows a significantly higher frequency with respect to positive (*p* < 0.05, 95% CI [0.26, 0.97]) and negative symptoms (*p* < 0.05, 95% CI [0.12, 0.85]) on the CAPE questionnaire. On the PANSS scale (assessed only in PD) individuals in cluster 1 show significantly higher negative symptoms (*p* < 0.01, 95% CI [3.47, 9.57]), higher suspiciousness (*p* < 0.05, 95% CI [0.24, 1.80]), more emotional withdrawal (*p* < 0.01, 95% CI [0.82, 2.34]) and social avoidance (*p* < 0.05, 95% CI [0.33, 2.67]) in comparison to individuals in cluster 2 (Fig. 2b, c; Table 2).

## Discussion

The current study investigated to which extent longitudinal trajectories of psychotic(-like) experiences in EMA are distinctive between outpatients diagnosed with psychotic disorders, healthy individuals, and healthy individuals with a first-degree relative with a psychotic disorder. Further, we investigated whether unsupervised machine learning can identify a subgroup of individuals potentially vulnerable to relapse to psychotic symptoms. Our analysis revealed two clusters which differ in their mean psychotic ratings but do not differ in their within-subjects rating variance across the EMA rating period. Cluster 1 consists mainly of PD, shows high mean EMA symptom ratings and high burden with respect to psychotic symptoms while cluster 2 shows lower symptoms and contains all study groups (Fig. 2).

The EMA trajectory clusters, which are based on EMA ratings of psychotic(-like) experiences, correspond to standard cross-sectional PANSS ratings. PD in cluster 1 show high EMA symptom ratings together with high PANSS positive and negative symptoms. PD in cluster 2 experience ‘minimal’ symptoms on the cross-sectional ratings which are reflected in lower (healthy-like) levels on the EMA rating scale and therefore these patients were grouped together with HC. We observe a similar relationship between EMA symptom ratings and cross-sectional ratings reported in the CAPE questionnaire. Taken together, this indicates that individuals with a psychotic disorder also report symptoms in phases where symptomatic burden is high [3]. EMA shows sensitivity to identify PD with high burden in symptoms using bottom-up ML approaches. In critical windows of high symptom ratings PD in cluster 1 might particularly profit from exchange with a clinical practitioner and a support team. This proof-of-concept study underlines the usefulness of EMA combined with advanced statistical methods as a digital healthcare device for monitoring symptoms post-hospitalization [3, 8, 9].

In cluster 2 which is characterized by low mean EMA ratings, a considerable proportion of PD (78% of the total sample of PD) are grouped together with most of the HC and RE. This suggests that most PD in cluster 2 might be clinically stable and outside of phases of acute psychosis. However, we find relatively high variance in the cross-sectional PANSS and CAPE scores in cluster 2 and individual scores of cluster 2 overlap with scores of individuals in cluster 1. Some individuals with high persecutory ideas and negative symptoms on the PANSS are assigned to cluster 2, which suggests that despite their symptom manifestation in the clinical examination, they did not experience symptoms within the EMA sampling period [1]. Alternatively, it is possible that high persecutory ideas in these individuals render them unable to engage with the app due to the perception of its ‘monitoring’ nature [12]. Studies also report that assessment of negative symptoms via EMA is moderated by working memory related cognitive deficits [44]. Therefore, some of the cluster 2 individuals who show high negative symptoms might have particularly strong impairments in working memory preventing them from reporting their symptoms in EMA.

Our findings indicate no clear differentiation of RE from HC across the investigated 7-day rating period as the longitudinal clustering approach does not reveal a cluster characterized by mainly RE. Most of the RE in cluster 2 show ratings comparable to HC ranging between 1 (“not at all”) and 4 (“neutral”) while only one participant indicates psychotic(-like) experiences on rare occasions (supplementary Fig S4). Longitudinal EMA trajectories as analyzed here do not show characteristics specifically distinguishing RE from HC. In contrast to studies with a priori group assignment, this suggests differentiation of groups with genetic risk for psychosis from HC based solely on EMA ratings is not straightforward in a situation where study group assignments are unknown [6]. However, as the current sample consists only of a low number of RE, replication in larger samples is crucial to validate these findings.

The current study has several limitations. First, we can not directly observe the likelihood of experiencing a relapse in psychotic symptoms for individuals assigned to the high symptomatology cluster (cluster 1) as we only have a clinical symptom assessment prior to the EMA assessment. Future research should investigate the validity of data-driven clusters in EMA to predict clinical outcomes such as relapse in symptoms. Second, due to the low sample size (especially regarding RE) potential biases related to the experimental implementation of EMA or sample specific characteristics might have influenced the clustering procedure and cluster structure. However, using repeated sampling of the current data clusters show good internal validity as indicated by stable cluster assignments across subsets of the data (Fig. 1c, supplementary material). PD in DECOP and SMARTAPP sample differ with respect to several clinical characteristics and cluster assignments were different across samples (supplementary material). However, individuals in both studies were prompted with the same EMA items regarding paranoia and hallucinations and time windows of EMA ratings between studies were harmonized. Therefore, differences in cluster assignments across samples rather represent differing recruitment strategies than a bias due to different EMA procedures. In sum, to show the generalizability of our findings in this proof-of-concept study, replication and validation in a larger sample is crucial. Third, due to the requirement of a complete data set for clustering, missing values had to be imputed. This resulted in a relatively high number of imputed values in the SMARTAPP sample (supplementary Fig S1). Finally, with the generation of well-validated models to detect individuals who are potentially in need of professional help new ethical and privacy challenges arise. If EMA finds more widespread application in clinical practice, it is important to work out guidelines of action for medical and psychological professionals in case an individual is detected to be in a potentially critical psychological condition [45]. The large-scale collection of highly sensible health data through EMA also requires explicit regulation to protect the privacy of the users from misconduct. However, critical information should be made easy to use and understand to the responsible health professionals [46]. As EMA has certain technological and infrastructural requirements, potentials needs of individuals, e.g., in areas with unstable wireless networks or with less technological expertise, should be anticipated [47].

## Conclusion

EMA allows to characterize symptom course and dynamics in daily life contexts and increasing evidence supports its feasibility as a reliable and valid tool for remote assessment of (psychotic) symptoms [3, 9]. The present proof-of-concept study investigated the usefulness of combining EMA with unsupervised machine learning in separating individuals who are potentially vulnerable to relapse into psychosis from those that appear more stable based on their longitudinal patterns of psychotic experiences. We identify one cluster of mainly PD, showing relatively high psychotic symptom level, which suggests that those could profit from direct engagement with professional services. The second cluster consists of the majority of PD and healthy individuals and showed a more healthy-like course of psychotic experiences which does not distinguish between healthy individuals with and without genetic burden. If findings can be replicated, the widespread application of EMA might deliver valuable information for clinicians and individuals with lived experience of psychosis to monitor symptoms in a low-cost, ecologically valid and high frequent manner [3, 8, 9]. Therefore, evidence from this study supports the implementation of digital healthcare devices in psychiatric and psychotherapeutic practice. 

## Supplementary Information

Below is the link to the electronic supplementary material.

Supplementary file1 (DOCX 29 KB)

Supplementary file2 (PDF 1893 KB)

## Data Availability

The data that support the findings of this study are available from the corresponding author, A-KF, upon reasonable request.

## References

[1] Oorschot M, Lataster T, Thewissen V, Bentall R, Delespaul P, Myin-Germeys I (2012) Temporal dynamics of visual and auditory hallucinations in psychosis. Schizophr Res 140:77–82. 10.1016/j.schres.2012.06.010 22784687 10.1016/j.schres.2012.06.010

[2] Oorschot M, Kwapil T, Delespaul P, Inez Myin-Germeys Oorschot M, Kwapil TR, Delespaul P, Myin-Germeys I (2009) Momentary Assessment Research in Psychosis. Am Phychol Assoc 616:498–505 10.1037/a0017077 19947784

[3] Harvey PD, Miller ML, Moore RC, Depp CA, Parrish EM, Pinkham AE (2021) Capturing Clinical Symptoms with Ecological Momentary Assessment: Convergence of Momentary Reports of Psychotic and Mood Symptoms with Diagnoses and Standard Clinical Assessments. Innov Clin Neurosci 18:24–30 34150360 PMC8195558

[4] Kimhy D, Delespaul P, Corcoran C, Ahn H, Yale S, Malaspina D (2006) Computerized experience sampling method (ESMc): Assessing feasibility and validity among individuals with schizophrenia. J Psychiatr Res 40:221–230. 10.1016/j.jpsychires.2005.09.007 16300791 10.1016/j.jpsychires.2005.09.007 PMC2992983

[5] Stamate D, Katrinecz A, Stahl D, Verhagen SJW, Delespaul PAEG, Van OJ, Guloksuz S (2019) Identifying psychosis spectrum disorder from experience sampling data using machine learning approaches. Schizophr Res 209:156–163. 10.1016/j.schres.2019.04.028 31104913 10.1016/j.schres.2019.04.028

[6] Feller C, Ilen L, Eliez S, Schneider M (2021) Psychotic experiences in daily-life in adolescents and young adults with 22q11. 2 deletion syndrome: An Ecological Momentary Assessment study. Schizophr Res 238:54–61 34607254 10.1016/j.schres.2021.09.024

[7] Verhagen SJW, Hasmi L, Drukker M, Van OJ, Delespaul PAEG (2016) Use of the experience sampling method in the context of clinical trials. Evid Based Ment 19:86–89 10.1136/ebmental-2016-102418 PMC5040762 27443678

[8] de Thurah L, Kiekens G, Sips R, Teixera A, Kasanova Z, Myin-germeys I (2023) Using Experience Sampling Methods to support clinical management of psychosis: The perspective of people with lived experience. Psychiatry Res 324:115207. 10.1016/j.psychres.2023.115207 37087818 10.1016/j.psychres.2023.115207

[9] van Os J, Verhagen S, Marsman A, Peeters F, Bak M, Marcelis M, Drukker M, Reininghaus U, Jacobs N, Lataster T, Simons C, ESM-MERGE-Investigators, Lousberg R, Gülöksüz S, Leue C, Groot PC, Viechtbauer W, Delespaul P (2017) The experience sampling method as an mHealth tool to support self-monitoring, self-insight, and personalized health care in clinical practice Richel Lousberg PhD 1. Depress Anxiety 34:481–493 28544391 10.1002/da.22647

[10] Van Os J, Linscott RJ, Myin-Germeys I, Delespaul P, Krabbendam L (2009) A systematic review and meta-analysis of the psychosis continuum: Evidence for a psychosis proneness-persistence-impairment model of psychotic disorder. Psychol Med 39:179–195. 10.1017/S0033291708003814 18606047 10.1017/S0033291708003814

[11] Alvarez-Jimenez M, Priede A, Hetrick SE, Bendall S, Killackey E, Parker AG, McGorry PD, Gleeson JF (2012) Risk factors for relapse following treatment for first episode psychosis: A systematic review and meta-analysis of longitudinal studies. Schizophr Res 139:116–128. 10.1016/j.schres.2012.05.007 22658527 10.1016/j.schres.2012.05.007

[12] Sablier J, Stip E, Jacquet P, Giroux S, Pigot H, Bouchard F, Marcotte N, Viboud JP, Bentaleb LA, Landry P, Lipp O, Tranulis C, Villeneuve M, Cloutier C, Lalancette C, Prince A, Vincent P, Lum M, Berrube MC, Lucas M, Boisset G, Guida M, Mazuire J, Meylan F, Meynier J, Pelletier G, Sportiello S, Doré-Gauthier V, Guévremont C, Nadeau-Marcotte F, Franck N (2012) Ecological assessments of activities of daily living and personal experiences with mobus, an assistive technology for cognition: A pilot study in schizophrenia. Assist Technol 24:67–77. 10.1080/10400435.2012.659324 22876729 10.1080/10400435.2012.659324

[13] Rössler W, Riecher-Rössler A, Angst J, Murray R, Gamma A, Eich D, van Os J, Gross VA (2007) Psychotic experiences in the general population: A twenty-year prospective community study. Schizophr Res 92:1–14. 10.1016/j.schres.2007.01.002 17363221 10.1016/j.schres.2007.01.002

[14] Linszen M, de Boer J, Schutte M, Begemann M, de Vries J, Koops S, Blom RE, Bohlken MM, Heringa SM, Blom JD, Sommer IEC (2022) Occurrence and phenomenology of hallucinations in the general population: A large online survey. Schizophr. 10.1038/s41537-022-00229-9 10.1038/s41537-022-00229-9 PMC9261095 35853871

[15] Nuevo R, Chatterji S, Verdes E, Naidoo N, Arango C, Ayuso-Mateos JL (2012) The continuum of psychotic symptoms in the general population: A cross-national study. Schizophr Bull 38:475–485. 10.1093/schbul/sbq099 20841326 10.1093/schbul/sbq099 PMC3329982

[16] Rössler W, Ajdacic-Gross V, Haker H, Rodgers S, Müller M, Hengartner MP (2015) Subclinical psychosis syndromes in the general population: Results from a large-scale epidemiological survey among residents of the canton of Zurich, Switzerland. Epidemiol Psychiatr Sci 24:69–77. 10.1017/S2045796013000681 24280150 10.1017/S2045796013000681 PMC6998132

[17] Fusar-Poli P, Borgwardt S, Bechdolf A, Addington J, Riecher-Rössler A, Schultze-Lutter F, Keshavan M, Wood S, Ruhrmann S, Seidman LJ, Valmaggia L, Cannon T, Velthorst E, De Haan L, Cornblatt B, Bonoldi I, Birchwood M, McGlashan T, Carpenter W, McGorry P, Klosterkötter J, McGuire P, Yung A (2013) The psychosis high-risk state: A comprehensive state-of-the-art review. Arch Gen Psychiatry 70:107–120. 10.1001/jamapsychiatry.2013.269 10.1001/jamapsychiatry.2013.269 PMC4356506 23165428

[18] Permanasari Y, Harahap E, Prayoga Ali E (2019) Speech recognition using Dynamic Time Warping (DTW). J Phys Conf Ser. 10.1088/1742-6596/1366/1/012091

[19] Hebbrecht K, Stuivenga M, Birkenhäger T, Morrens M, Fried EI, Sabbe B, Giltay EJ (2020) Understanding personalized dynamics to inform precision medicine : a dynamic time warp analysis of 255 depressed inpatients. BMC Med 18:1–15 33353539 10.1186/s12916-020-01867-5 PMC7756914

[20] Fett AKJ, Hanssen E, Eemers M, Peters E, Shergill SS (2022) Social isolation and psychosis : an investigation of social interactions and paranoia in daily life. Eur Arch Psychiatry Clin Neurosci 272:119–127. 10.1007/s00406-021-01278-4 34129115 10.1007/s00406-021-01278-4 PMC8803722

[21] Hanssen E, Balvert S, Oorschot M, Borkelmans K, Van OJ, Delespaul P, Fett A (2020) An ecological momentary intervention incorporating personalised feedback to improve symptoms and social functioning in schizophrenia spectrum disorders. Psychiatry Res 284:112695 31831201 10.1016/j.psychres.2019.112695

[22] Thewissen V, Bentall RP, Lecomte T, van Os J, Myin-Germeys I (2008) Fluctuations in Self-Esteem and Paranoia in the Context of Daily Life. J Abnorm Psychol 117:143–153. 10.1037/0021-843X.117.1.143 18266492 10.1037/0021-843X.117.1.143

[23] Oorschot M, Lataster T, Thewissen V, Lardinois M, Van Os J, Delespaul PAEG, Myin-Germeys I (2012) Symptomatic remission in psychosis and real-life functioning. Br J Psychiatry 201:215–220. 10.1192/bjp.bp.111.104414 22743843 10.1192/bjp.bp.111.104414

[24] Myin-Germeys I, Delespaul P, Van Os J (2005) Behavioral sensitization to daily life stress in psychosis. Psychol Med 35:733–741. 10.1017/S0033291704004179 15918350 10.1017/s0033291704004179

[25] Delespaul P, DeVries M, van Os J (2002) Determinants of occurence and recovery from hallucinations in daily life. Soc Psychiatry Psychiatr Epidemiol 37:97–104. 10.1007/s001270200000 11990012 10.1007/s001270200000

[26] Hanssen E, Krabbendam L, Robberegt S, Fett AK (2020) Social and non-social reward learning reduced and related to a familial vulnerability in schizophrenia spectrum disorders. Schizophr Res 215:256–262. 10.1016/j.schres.2019.10.019 31753593 10.1016/j.schres.2019.10.019

[27] Hanssen E, van Buuren M, Van Atteveldt N, Lemmers-Jansen ILJ, Fett AKJ (2022) Neural, behavioural and real-life correlates of social context sensitivity and social reward learning during interpersonal interactions in the schizophrenia spectrum. Aust N Z J Psychiatry 56:59–70. 10.1177/00048674211010327 34006142 10.1177/00048674211010327 PMC8721616

[28] Delespaul PAEG (1995) Assessing Schizophrenia in Daily Life: The Experience Sampling Method. University of Maastricht press, Maastricht

[29] Rintala A, Wampers M, Myin-Germeys I, Viechtbauer W (2019) Response compliance and predictors thereof in studies using the experience sampling method. Psychol Assess 31:226–235 30394762 10.1037/pas0000662

[30] Eisele G, Vachon H, Lafit G, Kuppens P, Houben M, Myin-Germeys I, Viechtbauer W (2022) The Effects of Sampling Frequency and Questionnaire Length on Perceived Burden, Compliance, and Careless Responding in Experience Sampling Data in a Student Population. Assessment 29:136–151. 10.1177/1073191120957102 32909448 10.1177/1073191120957102

[31] Myin-Germeys I, Oorschot M, Collip D, Lataster J, Delespaul P, Van Os J (2009) Experience sampling research in psychopathology: Opening the black box of daily life. Psychol Med 39:1533–1547. 10.1017/S0033291708004947 19215626 10.1017/S0033291708004947

[32] Kay SR, Fiszbein A, Opler LA (1987) The positive and negative syndrome scale (PANSS) for schizophrenia. Schizophr Bull 13:261–276. 10.1093/schbul/13.2.261 3616518 10.1093/schbul/13.2.261

[33] Stefanis NC, Hanssen M, Smirnis NK, Avramopoulos DA, Evdokimidis IK, Stefanis CN, Verdoux H, Van Os J (2002) Evidence that three dimensions of psychosis have a distribution in the general population. Psychol Med 32:347–358 11866327 10.1017/s0033291701005141

[34] Stekhoven DJ, Peter B (2012) MissForest - nonparametric missing value imputation for mixed-type data. Bioinformatics 28:112–118 22039212 10.1093/bioinformatics/btr597

[35] Stekhoven DJ (2013) missForest: Nonparametric Missing Value Imputation using Random Forest. R package version 1.4. https://cran.r-project.org/web/packages/missForest/missForest.pdf

[36] Eckert A (2018) parallelDist: Parallel Distance Matrix Computation using Multiple Threads. R package version 0.2.4. https://cran.r-project.org/package=parallelDist

[37] Kolde R (2019) pheatmap: Pretty Heatmaps. R package version 1.0.12. https://cran.r-project.org/package=pheatmap

[38] Sakoe H, Chiba S (1978) Dynamic Programming Algorithm Optimization for Spoken Word Recognition. IEEE Trans Acoust 26:43–49

[39] James G, Witten D, Hastie T, Tibshirani R (2021) An Introduction to Statistical Learning, 2nd edn. Springer, US, New York

[40] Hennig C (2007) Cluster-wise assessment of cluster stability. Comput Stat Data Anal 52:258–271. 10.1016/j.csda.2006.11.025

[41] Hennig C (2020) fpc: flexible procedures for clustering (Version 2.2–9)

[42] Jaccard P (1908) Nouvelles Recherches Sur La Distribution Florale. Bull la Société vaudoise des Sci Nat 44:223–270

[43] Benjamini Y, Hochberg Y (1995) Controlling the False Discovery Rate: A Practical and Powerful Approach to Multiple Testing. J R Stat Soc Ser B 57:289–300. 10.1111/j.2517-6161.1995.tb02031.x

[44] Moran EK, Culbreth AJ, Barch DM (2017) Ecological Momentary Assessment of Negative Symptoms in Schizophrenia: Relationships to Effort Based Decision Making and Reinforcement Learning. J Abnorm Psychol 126:96–105. 10.1037/abn0000240 27893230 10.1037/abn0000240 PMC5433621

[45] Wenze SJ, Miller IW (2010) Use of ecological momentary assessment in mood disorders research. Clin Psychol Rev 30:794–804. 10.1016/j.cpr.2010.06.007 20619520 10.1016/j.cpr.2010.06.007

[46] Ferguson SG, Jahnel T, Elliston K, Shiffman S (2020) Ambulatory Assessment. In: Wright A, Hallquist M (eds) The Cambridge Handbook of Research Methods in Clinical Psychology. Cambridge University Press, pp 301–311

[47] Trull TJ, Ebner-Priemer U (2013) Ambulatory Assessment. Annu Rev Clin Psychol 9:151–176. 10.1146/annurev-clinpsy-050212-185510 23157450 10.1146/annurev-clinpsy-050212-185510 PMC4249763

