# Correction to: Ecological momentary assessment (EMA) combined with unsupervised machine learning shows sensitivity to identify individuals in potential need for psychiatric assessment

**DOI:** 10.1007/s00406-024-01939-0

**Published:** 2024-11-15

**Authors:** Julian Wenzel, Nils Dreschke, Esther Hanssen, Marlene Rosen, Andrej Ilankovic, Joseph Kambeitz, Anne-Kathrin Fett, Lana Kambeitz-Ilankovic

**Affiliations:** 1 Department of Psychiatry and Psychotherapy, Faculty of Medicine and University Hospital of Cologne, University of Cologne, Cologne, Germany; 2 Hersencentrum Mental Health Institute, Amsterdam, The Netherlands; 3 Department of Psychiatry, Faculty of Medicine, University of Belgrade, Belgrade, Serbia; 4 Department of Psychology, City St George’s, University of London, London, United Kingdom; 5 Department of Psychosis Studies, Institute of Psychology, Psychiatry and Neuroscience, London, United Kingdom; 6 Department of Psychology, Faculty of Psychology and Educational Sciences, Ludwig-Maximilian University, Munich, Germany

Upon an exchange with an expert researcher in the field of dynamic time warping (DTW) we implemented an additional step (‘z-normalization’) in the preprocessing pipeline of our ecological momentary assessment (EMA) data. This step is required in DTW analysis to capture similarities between temporal dynamics, i.e. similarities in the *shape*, rather than similarities in the *absolute (mean) rating* in the EMA trajectories. Even small differences in the scale or offset will reduce any similarity information encoded in the dynamic or shape of this trajectory. As an example, when using DTW we want to be able to recognize both 7 and _7_, despite their differences in size (scale), similarly, as we want to be able to recognize ^7^ and _7_, despite different offsets. Z-normalization of the EMA trajectories before applying DTW is essential as it removes differences in mean ratings between EMA trajectories and in this way identifies differences in rating dynamics [1]. Furthermore, it was necessary to adapt the clustering algorithm in order to be able to use distance matrices for clustering.

We adapted the following aspects of the original pipeline:

1. We implemented the aforementioned z-normalization as part of the preprocessing prior to application of DTW and the clustering algorithm.
2. We adjusted the selection of the clustering algorithms to better fit the data and investigated the results with a hierarchical as well as the PAM (partitioning around medoids) clustering algorithm. We compared several cluster indices to decide which number of clusters was the most optimal for the data set.

In the new analysis the PAM clustering algorithm also reveals a two-cluster solution as most stable (Jaccard indices: cluster 1 = 0.77; cluster 2 = 0.88). However, in contrast to previous results the cluster indices regarding the cluster number were inconsistent and did not indicate a clear number of clusters. In line with our previous results, one cluster (cluster 2) shows higher mean EMA symptom ratings than the other (cluster 1). Cluster 2 had significantly higher ratings on cross-sectional Positive and Negative Syndrome Scale (PANSS) ratings of general symptoms than cluster 1. However, the new cluster solution shows no differences on other PANSS scales and the self-report Community Assessment of Psychic Experience (CAPE) questionnaire (Fig. 1; Table 1).

This study included three study groups in the clustering, i.e. outpatients diagnosed with a psychotic disorder (PD), healthy individuals (HC), and healthy individuals with a first-degree relative with psychosis (RE). All three study groups were represented in both clusters while most of PD (73%, *N* = 40) and RE (70%, *N* = 14) were assigned to cluster 2. We find no clear differentiation of RE from HC or PD in clusters across the investigated 7-day EMA rating period.

**Fig. 1:**
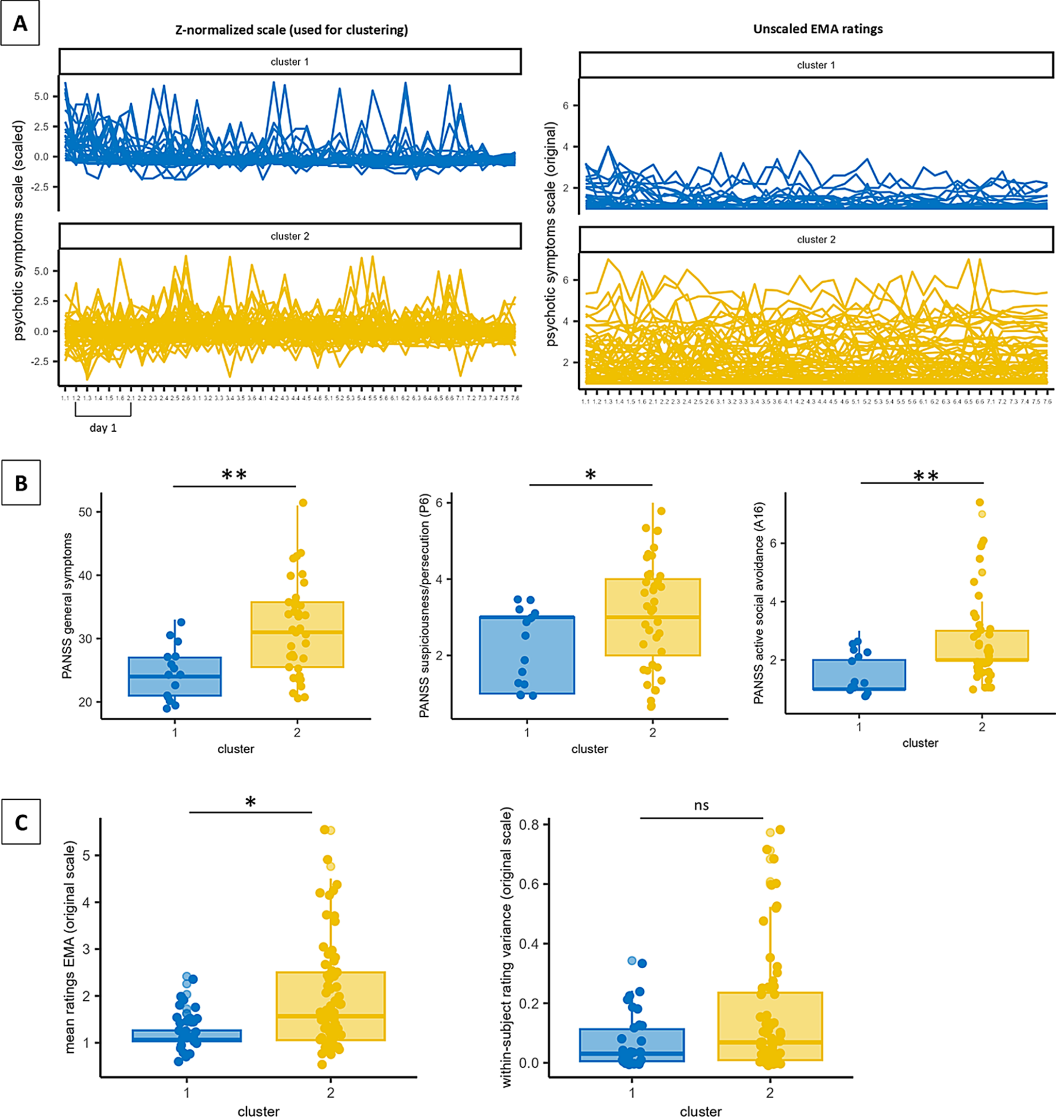
Cluster characteristics. (**A**) We obtained a two-cluster solution with distinct characteristics in their EMA ratings. The Z-normalized EMA psychotic symptom scale data is shown in the left and the unscaled, i.e. raw data is shown in the right panel. Z-normalized psychotic symptom scores were used for clustering. (**B**) Clusters showed significantly different clinical scores on the PANSS general symptom scale (left), as well as on two of the individual PANSS items, suspiciousness (middle) and active social avoidance (right). (**C**) Additionally, the revised (z-normalized) clusters 1 and 2 showed significantly different mean EMA ratings of psychotic symptoms (left panel) but no significant difference with respect to within-subject EMA rating variance of these (right panel). Significances: * *p* < 0.05, ** *p* < 0.01, *** *p* < 0.001

**Table 1:** Demographic and clinical cluster characteristics

	Cluster 1 (*N* = 34)	Cluster 2 (*N* = 66)	t value/chi^2^, F-value	*p*_fdr_
**study group** ^**a**^	15/6/13	40/14/12	4.861	0.704
**age**,** mean (sd)**	37.08 (9.31)	39.22 (11.58)	-0.998	0.830
**sex = female (%)**	11 (39)	28 (74)	0.580	0.830
**educational status** ^**b**^	13/10/6/0/0/2	16/11/10/2/2/2	0.623	0.830
**medication**,** n (%)**^**c**^				
antipsychotic	14 (100)	35 (95)	0.788	0.830
antidepressant	3 (23)	12 (32)	0.401	0.838
benzodiazepine	0 (0)	5 (16)	1.806	0.830
mood stabilizers	0 (0)	1 (3)	0.331	0.999
**PANSS**				
positive symptoms, mean (sd)	11.73 (3.65)	15.13 (5.77)	-2.570	0.061
negative symptoms, mean (sd)	12.40 (5.25)	16.36 (5.37)	-2.467	0.076
general symptoms, mean (sd)	24.67 (4.37)	31.50 (7.28)	-4.182	**0.003**
**PANSS (individual items)**				
delusions (P1)	2.00 (1.25)	2.44 (1.37)	-1.114	0.510
hallucinatory behavior (P3)	2.13 (1.60)	2.79 (1.70)	-1.338	0.416
suspiciousness/persecution (P6)	2.20 (0.94)	3.12 (1.45)	-2.758	**0.047**
emotional withdrawal (N2)	1.93 (1.22)	2.51 (1.35)	-1.513	0.334
active social avoidance (A16)	1.53 (0.74)	2.64 (1.48)	-3.636	**0.004**
**CAPE** ^**d**^				
positive symptoms - freq, mean (sd)	1.47 (0.38)	1.65 (0.52)	1.543	0.435
positive symptoms - dis, mean (sd)	1.81 (0.61)	2.00 (0.71)	0.373	0.735
negative symptoms - freq, mean (sd)	1.72 (0.38)	1.97 (0.59)	2.395	0.326
negative symptoms - dis, mean (sd)	1.86 (0.61)	2.18 (0.61)	3.668	0.169
depressive symptoms - freq, mean (sd)	1.89 (0.55)	1.98 (0.62)	0.382	0.735
depressive symptoms - dis, mean (sd)	2.46 (0.69)	2.46 (0.66)	0.067	0.863

a: numbers correspond to PD/RE/HC

b: numbers correspond to university/college/secondary school/primary school/other/none

c = information on medication is based on PD individuals

d = F- and p-values indicate the main effect for cluster; there were no significant interactions present

Abbreviations: PANSS = Positive and Negative Syndrome Scale; CAPE = Community Assessment of Psychic Experience; freq = frequency; dis = distress; sd = standard deviation. Significant p values are in bold

Due to the lack of z-normalization of our data in the original manuscript, DTW and therefore the clustering solution was skewed towards capturing differences between mean ratings of individuals and not as intended, capturing differences in the rating dynamics (‘shape’) of individuals. In the original results this was indicated by the finding that individuals assigned to cluster 1 show significantly higher mean average symptom ratings as compared to individuals assigned to cluster 2. In contrast, rating variance did not significantly differentiate the clusters. The high mean rating cluster (cluster 1) in return corresponded to the significantly higher cross-sectional ratings reported on the PANSS and CAPE questionnaire.

After applying z-standardization, (1) cluster indices and cluster stability were inconsistent with respect to the optimal cluster solution for the current data and (2) the unsupervised machine learning did no longer identify individuals with distinct clinical characteristics in terms of positive and negative symptoms on the PANSS or CAPE. However, in contrast to our previous analysis we now found that individuals assigned to cluster 2 experienced higher general psychopathology. As for our previous analysis, differences on the single PANSS items of suspiciousness and active social avoidance emerged between the two clusters. Individuals in cluster 2 were further characterized by a specific pattern in their EMA ratings. However, as visible in Fig. 1C, it is likely that this effect was still driven by differences in the absolute mean EMA ratings and to a lesser extent by the rating dynamics.

That is, in contrast to the original manuscript, we now need to conclude that the correspondence between positive and negative symptom ratings on cross-sectional clinical interview and questionnaire measures and the EMA rating dynamics, i.e. the shape and pattern of individual EMA ratings over time, is relatively low. However, we need to acknowledge that even after removing mean differences we still find significant differences between clusters with respect to EMA mean symptom ratings. Thus, clustering of the standardized EMA rating dynamics, seems to be less informative with respect to the overall severity of symptoms, as indicated in clinical interviews or questionnaires before the EMA rating period.

In sum, our findings with z-standardized data do not support many of our original conclusions, which suggested that dynamics in EMA ratings correspond well to ratings of positive and negative symptoms in clinical assessments and questionnaires. However, they do not exclude the potential informativeness and clinical usefulness of characterizing dynamic patterns of EMA ratings, e.g. in relationship to relapse or to differences in perceived functional impairment of an individual which we did not investigate in the current study.

**Acknowledgements**: We would like to thank Prof. Eamonn Keogh (University of California – Riverside) who provided helpful comments and constructive feedback and who supported us in applying the corrections to our data analysis.
