# Supplementary material for: Ecological momentary assessment (EMA) combined with unsupervised machine learning shows sensitivity to identify individuals in potential need for psychiatric assessment
Source: Eur Arch Psychiatry Clin Neurosci. 2023 Sep 16;274(7):1639–49. doi: 10.1007/s00406-023-01668-w (PMC11422424; doi:10.1007/s00406-023-01668-w)
Supplement: Supplementary file 1 — Supplementary file1 (DOCX 29 KB) [file 406_2023_1668_MOESM1_ESM.docx]

**Title:**

Ecological momentary assessment (EMA) combined with unsupervised machine learning shows sensitivity to identify individuals in potential need for psychiatric assessment

**Authors:**

Julian Wenzel^1^, Nils Dreschke^1^, Esther Hanssen^2^, Marlene Rosen^1^, Andrej Ilankovic^3^, Joseph Kambeitz^1^, Anne-Kathrin Fett^4,5*^, Lana Kambeitz-Ilankovic^1,6*^

^*authors contributed equally to this work^

**Affiliations :**

1 Department of Psychiatry and Psychotherapy, Faculty of Medicine and University Hospital of Cologne, University of Cologne, Cologne, Germany

2 Hersencentrum Mental Health Institute, Amsterdam, The Netherlands

3 University of Belgrade, Faculty of Medicine, Department of Psychiatry, Serbia

4 Department of Psychology, City, University of London, London, UK

5 Institute of Psychiatry, Psychology and Neuroscience, Department of Psychosis Studies, King’s College London, London, UK

6 Faculty of Psychology and Educational Sciences, Department of Psychology, Ludwig-Maximilian University, Munich, Germany

**Corresponding author:**

Anne-Kathrin Fett

Department of Psychology, City, University of London, London, UK

anne-kathrin.fett@city.ac.uk

**Supplementary material**

**Results**

**Clustering results for different window sizes**

In order to account for different delays in ratings between participants we investigated Sakoe Chiba Band [1] window sizes ranging between 2 (small delay) and 16 (high delay) (supplementary Fig S2). Overall, varying the window size of the Sakoe Chiba Band, i.e. allowing for more or less stretching and compression, had no impact on the number of clusters identified and only minimal impact on the observations grouped within clusters. After resampling, a 2-cluster solution appeared as the optimal number of clusters for each window size. Pairwise comparisons between the 2-cluster solutions of each window sizes revealed a mean adjusted rand-index of 0.97, indicating high consistency of cluster assignments across the cluster solutions. For further cluster characterization, we used the results for a window size of two.

**Differences between samples**

In order to assess the comparability of the two samples we calculated t-tests on several demographic and clinical characteristics between PD of the SMARTAPP and the DECOP sample (table S1). We found significantly higher general PANSS score in PD of the SMARTAPP sample as compared to the DECOP sample (t(44.703) = -2.370, p < 0.05) and significantly higher frequency of positive symptoms based on the CAPE questionnaire (t(52.593) = 2.533, p < 0.05). We found that in the SMARTAPP sample a significantly lower amount of PD were assigned to the higher symptom cluster (cluster 1) (X^2^(1, N=55) = 4.305, p < 0.05). There were no differences in medication intake between samples for antipsychotic (X^2^(1, N=51) = 0.000, p > 0.05) and antidepressant medication (X^2^(1, N=51) = 0.187, p > 0.05), benzodiazepines (X^2^(1, N=51) = 3.051, p > 0.05) and mood stabilizers (X^2^(1, N=51) = 0.000, p > 0.05).

**Literature**

1. Sakoe H, Chiba S (1978) Dynamic Programming Algorithm Optimization for Spoken Word Recognition. IEEE Trans Acoust 26:43–49

**Supplementary tables**

**Table S1.** Comparison between samples used in the current study.

|  | **DECOP** | **SMARTAPP** | **Statistics** | |
| --- | --- | --- | --- | --- |
|  | N = 29 | N = 26 | T/X^2^ | p value |
| **age in years (sd)** | 39.07 (9.90) | 40.85 (10.89) | -0.632 | 0.531 |
| **female sex (%)** | 6 (20.70) | 10 (38.55) | 1.326 | 0.250 |
| **living status^a^** | 20/9/0 | 17/4/5 | 7.024 | 0.029 |
| **cluster assignment^b^** | 10/19 | 2/24 | 4.305 | 0.038 |
| **diagnosis^c^** | 2/4/22 | 4/7/13 | 3.512 | 0.191 |
| **PANSS** |  |  |  |  |
| postive (sd) | 13.00 (4.21) | 15.46 (6.38) | -1.660 | 0.104 |
| negative (sd) | 15.21 (5.82) | 15.30 (5.43) | -0.061 | 0.952 |
| general (sd) | 27.33 (5.66) | 31.88 (8.06) | -2.370 | 0.022 |
| **CAPE freq** |  |  |  |  |
| positive (sd) | 1.890 (0.583) | 1.527 (0.478) | 2.533 | 0.014 |
| negative (sd) | 2.054 (0.583) | 1.911 (0.613) | 0.884 | 0.381 |
| depressiv (sd) | 2.073 (0.583) | 1.944 (0.754) | 0.707 | 0.483 |
| **CAPE dis** |  |  |  |  |
| positive (sd) | 2.297 (0.736) | 2.089 (0.620) | 1.082 | 0.285 |
| negative (sd) | 2.313 (0.668) | 2.278 (0.605) | 0.207 | 0.837 |
| depressiv (sd) | 2.534 (0.707) | 2.344 (0.691) | 1.001 | 0.319 |

^a = numbers correspond to the following living status: alone/family or partner/other^

^b = numbers correspond to the following clusters: cluster1/cluster2^

^c=numbers correspond to the following diagnoses: psychotic disorder/schizo-affective disorder/schizophrenia^

^Abbreviations: freq = frequency; dis = distress; sd = standard deviation.^
