# Supplementary material for: Ecological momentary assessment (EMA) combined with unsupervised machine learning shows sensitivity to identify individuals in potential need for psychiatric assessment
Source: Eur Arch Psychiatry Clin Neurosci. 2023 Sep 16;274(7):1639–49. doi: 10.1007/s00406-023-01668-w (PMC11422424; doi:10.1007/s00406-023-01668-w)
Supplement: Supplementary file 2 — Supplementary file2 (PDF 1893 KB) [file 406_2023_1668_MOESM2_ESM.pdf]

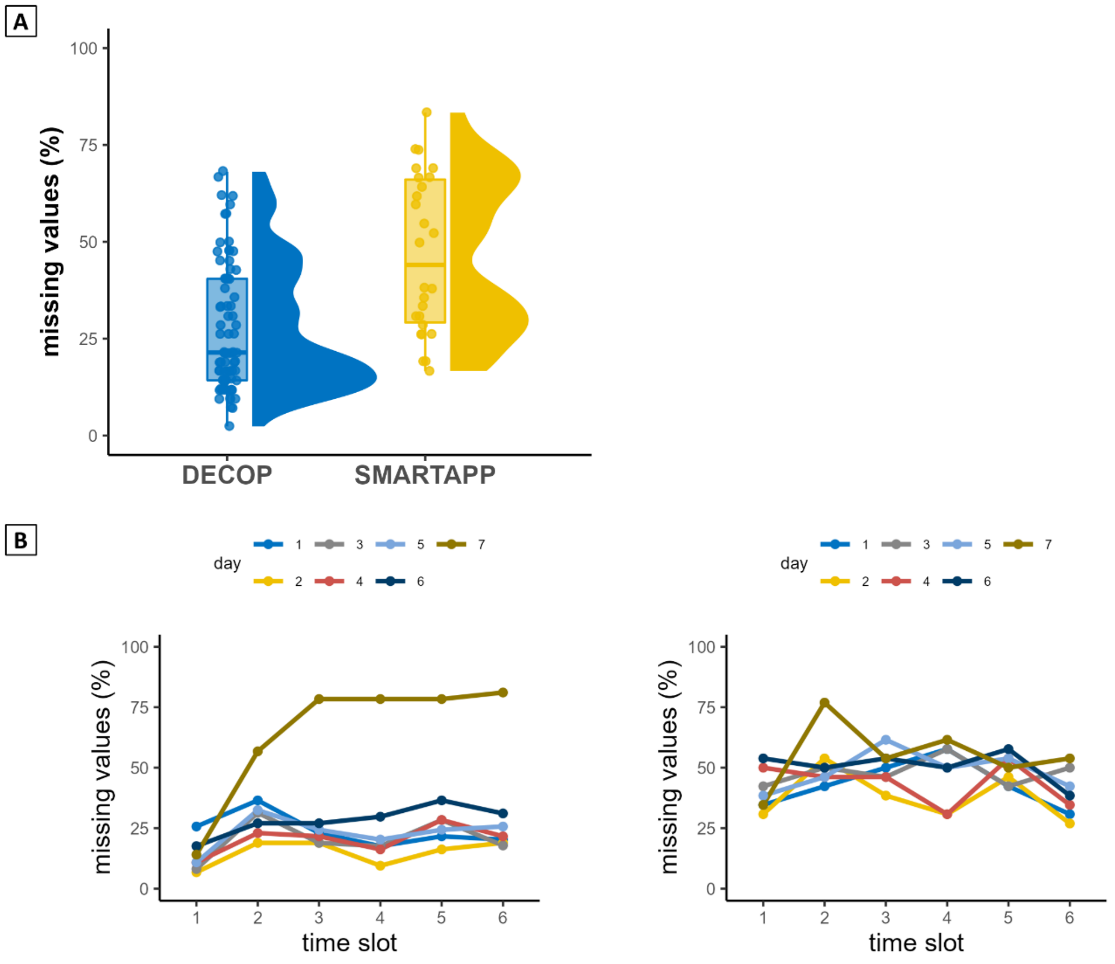

Figure S 1. Percentage of missing values of the current sample. The percentage of missing values for each participant for the five investigated EMA items (“I hear voices”, “I see things”, “I feel that others dislike me”, “I feel suspicious”, “I feel that others intend to harm me”) during the 7-day sampling period separate for each sample (A) and (B) the percentage of missing values for each time slot per day are given for both DECOP (left) and SMARTAPP (right) sample.

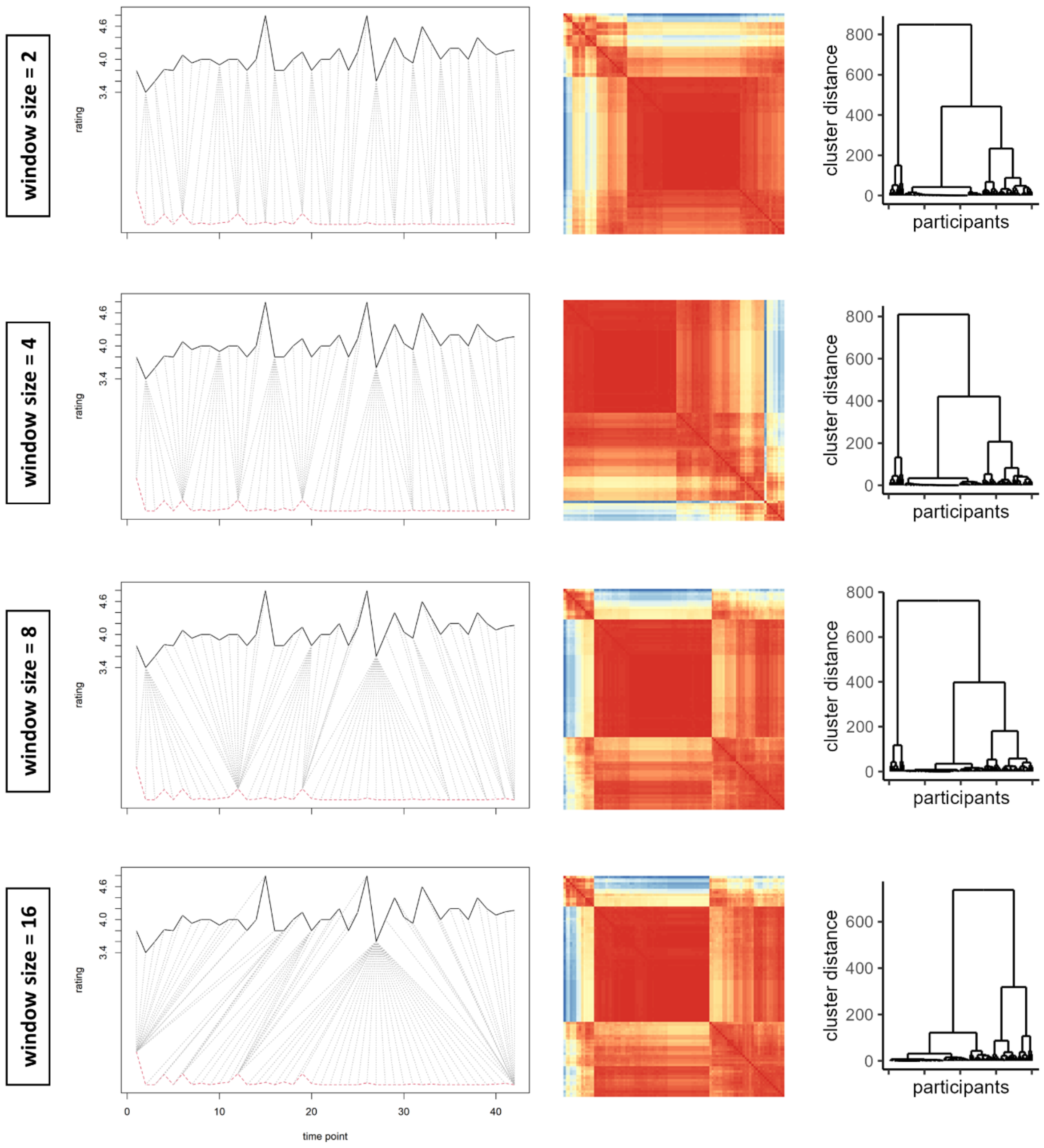

Fig S 2. Dynamic time warping, similarity matrix and dendrogram for different window sizes. The left column shows an example of the dynamic time warping process between two individuals in the sample for window sizes of two, four, eight and sixteen of the Sakoe Chiba Band. The middle and right column displays the similarity matrix and dendrogram for all individuals across the four window sizes. Varying the window size had no impact on the number of clusters identified and only minimal impact on the observations grouped within clusters.

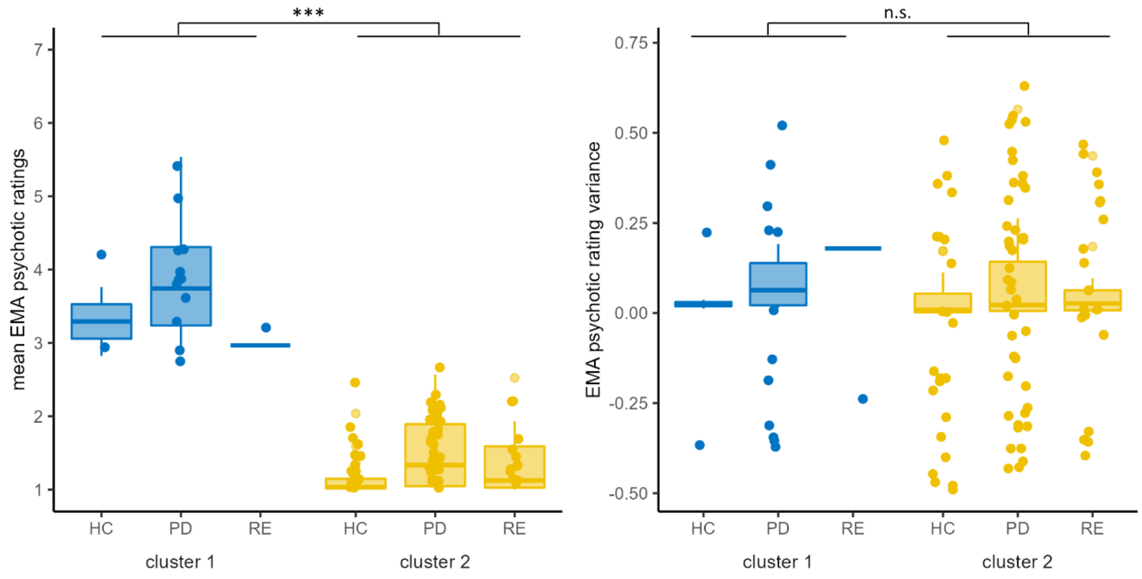

Fig S 3. EMA rating cluster characteristics. The left plot represents the mean EMA psychotic rating across the 7-day period per cluster and study group. The right plot represents the variance of the EMA psychotic ratings divided by the cluster specific mean EMA psychotic ratings over the 7-day period per cluster and study group. Abbreviations: PD = individual with psychosis spectrum disorder, HC = healthy control, RE = HC + first degree relative of individual with PD. Significances: * $p < 0.05$, ** $p < 0.01$, *** $p < 0.001$.

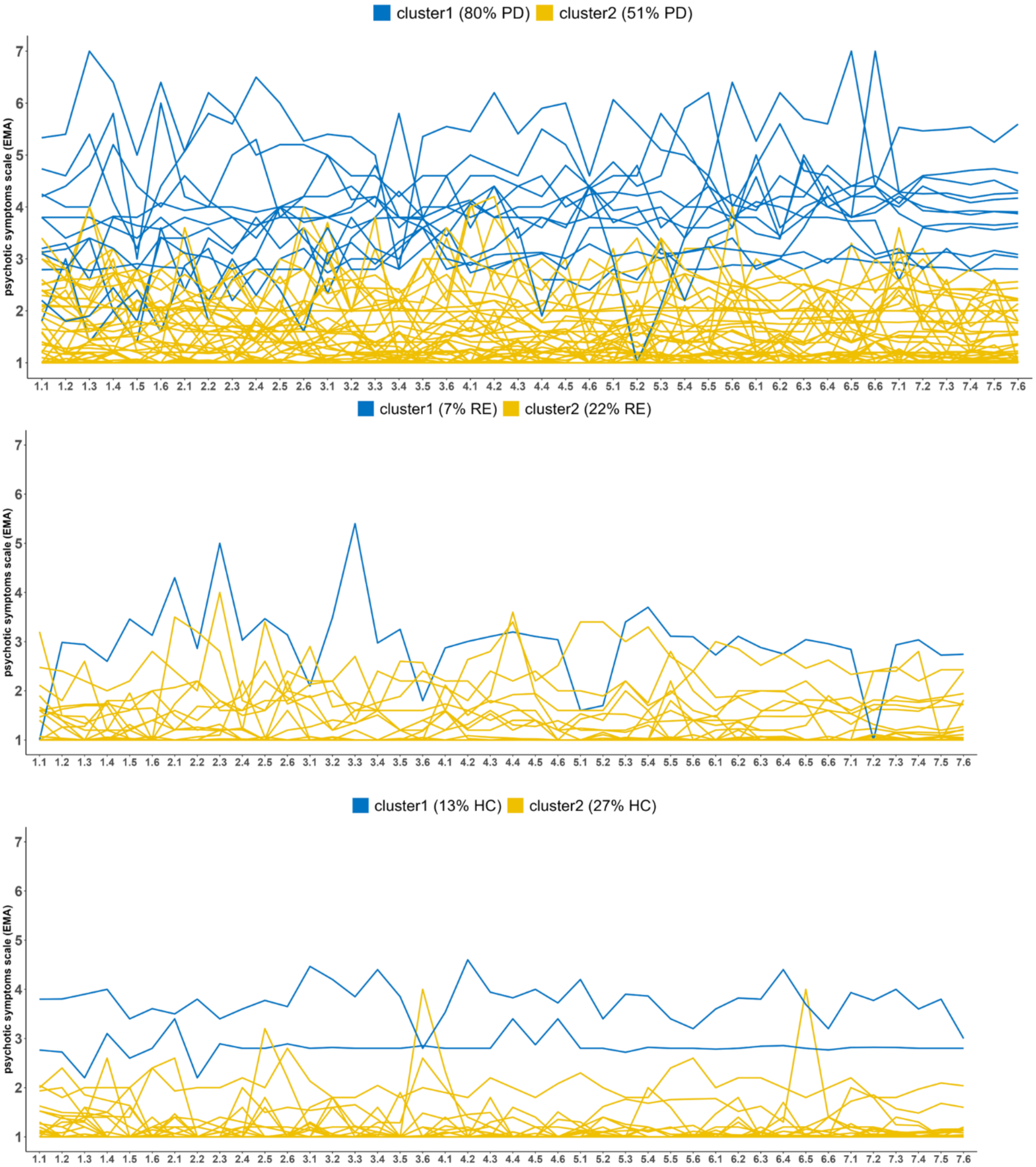

Figure S 4. EMA trajectories separated for study group and identified clusters. Abbreviations: PD = individuals with psychosis spectrum disorder, HC = healthy control, RE = HC + first degree relative of individual with PD.
